# Estimation of bimodal soil-water characteristics curve under wetting process

**DOI:** 10.1371/journal.pone.0325646

**Published:** 2025-06-25

**Authors:** Nura Bello, Alfrendo Satyanaga, Nurly Gofar, Jong Kim

**Affiliations:** 1 Department of Civil and Environmental Engineering, School of Engineering and Digital Sciences, Nazarbayev University, Nur-Sultan, Kazakhstan; 2 Department of Civil and Environmental Engineering, School of Engineering and Digital Sciences, Nazarbayev University, Nur-Sultan, Kazakhstan; 3 Department of Civil Engineering, Post Graduate Program, Universitas Bina Darma, Palembang, Indonesia; 4 Department of Civil and Environmental Engineering, School of Engineering and Digital Sciences, Nazarbayev University, Nur-Sultan, Kazakhstan; Jazan University College of Engineering, SAUDI ARABIA

## Abstract

Soil-water characteristics curve (SWCC) is hysteretic. The hysteretic curves represent the soil conditions during drying and wetting processes. Almost all failure of geotechnical structures, particularly in soils with dual porosity (bimodal soils) occurs during soil wetting, making the wetting curve more relevant to geotechnical applications than the drying curve. Still, due to the high-cost implications, time require, and difficulties associated with measuring SWCC under wetting process, the wetting curve has been continuously neglected. This leads to analyses that do not reflect the actual soil condition, which may result in structural failure. Although bimodal soils are encountered in many areas across the globe, there is no mathematical model for estimating bimodal SWCC under wetting process. This work developed a new mathematical equation to estimate a bimodal SWCC under wetting process. The proposed model also has parameters with physical meaning in relation to the variables of the wetting SWCC, which is very important in practical applications and numerical analyses. Six soil types were used in the model validation. The overall model performance has been evaluated through statistical methods using the coefficient of determination (R^2^) and Root Mean Square Error (RMSE). The evaluation also indicates a good performance of the model, with an average RMSE of 0.0263 and an average R^2^ of 0.9945 for the six soil samples used in the validation. Given the significance of wetting-induced failures, the proposed model is highly relevant in engineering applications.

## Introduction

Bishop and Blight [1] effective stress theory has indicated that soil moisture characterized by negative pore water pressure (suction) is the critical factor controlling soil behavior. A similar concept of effective stress for unsaturated soil is also proposed by Biot [2] in term of porous media mechanics. Geotechnical structures such as earth slopes and retaining walls are affected by the amount of moisture within the soil’s pores, because the significant portion of the earth on which those structures are built are actually partially saturated (unsaturated). However, the majority of past geotechnical analyses and designs were performed on the assumption that these soil pores are fully saturated. Soil-water Characteristics Curve (SWCC) is the most important unsaturated soil property that relates the amount of water in the soil and the soil suction [3–6]. SWCC has been used by many researchers to estimate other properties of unsaturated soil, such as permeability and shear strength of soil under unsaturated conditions, from which all other unsaturated soil behaviors are being modelled [3,7,8].

To estimate other important unsaturated soil properties with required accuracy and precision, the input of SWCC must be relevant, and reflect the actual soil condition. SWCC is hysteretic as reported by several researchers [9–11]. Hysteresis means there are always two boundary curves representing the drying and wetting conditions of the soil, and several other scanning curves in between the boundary curves. Due to hysteresis, at the same matric suction, moisture contents differ during soil drying and wetting [12]. As such, wetting SWCC reflects lower moisture content as compared to drying SWCC. This hysteresis phenomenon may be caused by any of the following key mechanisms.

a. Entrapped air bubbles occupying the soil pores, blocking equal moisture entering the pores during re-wetting at same matric suction [3,13,14].
b. Effects of meniscus contact angle which is wider when the soil is wetting, as against been narrower during drying. This translates into bigger and smaller radius of meniscus respectively, leading to less moisture content in wetting than in drying. [14–16].
c. Irregularity in cross-section of soil voids (Ink-bottle effect) which affects the movement of water. Pushing the moisture through the neck connecting the two pores with varying sizes requires higher suction during rewetting hence causing hysteresis [3,17].
d. An aged soil having a long history of swelling and shrinkage due to routine wetting & drying over time, and these causes hysteresis as well [18].

Although all the mentioned mechanisms contribute to the total hysteresis of SWCC, effect of entrapped air is more evident, as compared with the rest [19].

Studies were conducted in the past to model the hysteresis of SWCC. The hysteresis prediction models are categorized by Pham et al. [20] into two, the “domain models” and the “empirical models”. Domain models that were first used to describe the theory behind the concept of hysteresis includes Enderby [21] diagram, Preisach [22] diagram, and Neel [23,24] diagram. Moreover, other researchers such as Poulovassilis [25] and Mualem [26], used Néel [24] diagram to describes the hysteresis of SWCC. The domain models themselves are subdivided into “the dependent and independents” based on the influences of neighboring domains (group of pores) on each other [20].

On the other hand, there are also empirical models such as Feng and Fredlund [27]; Zhai et al. [28], Bello et al. [29] and Kawai et al. [30]. This category work by fitting laboratory observed hysteretic SWCC data points to an equation developed based on empirical parameters. Empirical models consist of two sub-groups as well. In the first sub-group, the same curve fitting equation is applied on both drying and wetting curves, and the values of the fitting parameters adjusted separately. While the other sub-group is composed of empirical models that relate to the drying and wetting curves based on specified points (variables) of both curves.

The soil mechanical behavior during wetting process is very important, because the majority of failures in earth structures occur during soil wetting. The term “wetting process” signifies the overall process in which water infiltrate dry soil and fill its empty pores until full saturation is achieved. This process is responsible for important changes in hydro-mechanical properties of soil. Therefore, the wetting SWCC is more relevant for practical purposes as compared to the drying SWCC. But researchers often ignore the wetting curve and perform unsaturated soil analyses with the drying curve even in situations requiring modelling soil wetting [31]. This is due to cost and difficulties associated with laboratory measurement of wetting SWCC [32]. However, ignoring hysteresis leads to analyses which does not reflects the actual soil condition [20,33]. This results in conducting conservative analyses that are uneconomical to achieve, or totally inaccurate, causing structural failures and possible loss of lives [34].

Apart from the cost and time required to generate complete hysteretic SWCC, the wetting curve is also difficult to measure. Moreover, representing these curves in a mathematical analysis involves a rigorous procedure [20]. Therefore, proposing a mathematical method to estimating the wetting curve with satisfactory accuracy will save cost and time. It will be useful in achieving soil analysis that are closer to field conditions. The estimation model should equally have physical meaning in relation to the variables of SWCC. This is because having parameters with no physical meaning is one of the major weakness of the available SWCC models in the literature [35].

The majority of the research on SWCC are concentrated on unimodal curves. These unimodal curves are obtained largely on well-graded soil [36–38]. But recent works have shown that soils with gap-graded grain size distribution (GSD) could give either unimodal or bimodal SWCC [35,39–41]. “Unimodal SCWCC” is single S-shaped sigmoidal curve while “bimodal SWCC” is a dual S-shaped sigmoidal curve. Several soil types with two dominant pore size family (micropores and macropores) such as residual soils, colluvial soils and soils used in transportation pavements usually show bimodal GSD and bimodal SWCC [35]. Clayey soils also exhibit such patterns due to dual pore sizes in pore size distributions during dewatering [42]. Soils that are characterized by bimodal GSD & bimodal SWCC are encountered in many natural formations across the globe, and their use in man-made earth structures is common. It is imperative then to consider this dual porosity nature when conducting analyses.

Natural and man-made earth structures are often encountered in an unsaturated part of the soil. Since SWCC is the most important property of the unsaturated soil, relevant SWCC representing actual soil conditions such as the nature of its grain size distribution, and hysteresis of its SWCC should always be used in an unsaturated soil’s analyses. Cost, time and difficulties associated with SWCC measurement under wetting process are the limitations hindering the use of complete SWCC hysteresis in numerical analyses.

The surge in conducting transient seepage analyses based on unsaturated soil mechanics entails the necessity for the use of actual data of moisture migration in the soil pores, at all conditions. The lack of faster and simpler methods to obtain wetting SWCC necessitates the use of drying SWCC, albeit accurate modelling of soil wetting could only be made with the wetting curve. These limitations would be entirely avoided if the wetting curve could be estimated with a required accuracy and simplicity. Although bimodal soils associated with bimodal SWCC are encountered in several locations, there is no developed mathematical equation to estimate bimodal SWCC under the wetting process. For this reason, this work proposed a mathematical model to estimate a bimodal wetting SWCC entirely from the bimodal drying SWCC, with required accuracy. All the parameters of the proposed model also have physical meaning.

## Equation for estimating Bimodal wetting SWCC

### Theory

**Fig 1:**
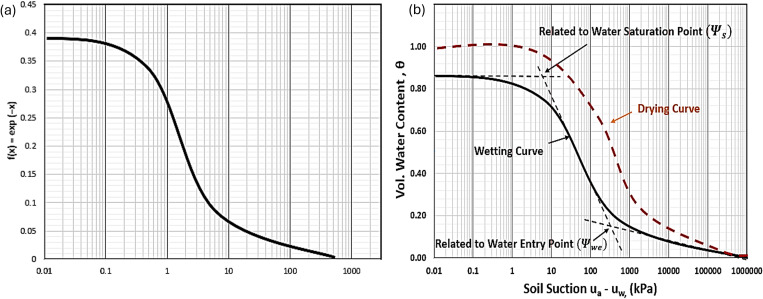
(a) Graph of exponential function. (b) Typical unimodal wetting SWCC.

Exponential function is an arithmetic expression widely uses in modelling physical phenomena in mathematics, physics and several other scientific applications [43]. Exponential function entails several forms, based on its nature of change, either growing or decaying exponentially. The graph of the function (Fig 1a) closely resembles the graph of SWCC (Fig 1b). SWCC is a sigmoidal semi-logarithmic graph, with moisture content (usually volumetric) on the arithmetic y-axis and the soil suction on the logarithmic x-axis from 0 kPa to 10^6^ kPa. In Fig (1b), y-axis represents the normalized volumetric water content $\left(\frac{\theta -\theta_{r}}{\theta_{s}-\theta_{r}}\right)$, while the x-axis is the suction $\left(u_{a}-u_{w}\right)$in log scale. The curves in the figure are the primary drying and wetting curves, while the drying curves begins at saturation point, the wetting curve usually does not fully recover the pore moisture due to largely the effects of entrapped air [12,44].

A general mathematical equation to model a unimodal SWCC utilizing the similarity between graph of exponential function and SWCC is given by equation (1).


$$\frac{\theta -\theta_{r}}{\theta_{s}-\theta_{r}}=exp[-t(\psi )\backslash ]$$ (1)

where (ψ) is the normalized suction between air entry value and residual suction $\frac{\psi -\psi_{a}}{\psi_{r}-\psi_{a}}$ of the drying curve.


$$\theta (\psi )=\;\left(\theta_{s}-\theta_{r}\right)\;\exp\;\left[-t\;\left(\frac{\psi -\psi_{a}}{\psi_{r}-\psi_{a}}\right)\right]+\;\theta_{r}\;\;$$ (2)

In terms of wetting SWCC, the variables of the curve would be used and the equation (2) become;


$$\theta (\psi )=\theta_{we}+{(\theta }_{ws}-\theta_{we})\;exp\left[-t\;\left(\frac{\psi \;-\;\psi_{ws}}{\psi_{we}-{\;\psi }_{ws}}\right)\right]$$ (3)

$$F(\psi )=0;\;when\;(\psi )\;<\;{(\psi }_{ws})$$ (4)

$$F(\psi )\;=\;exp\left[-t(\;\frac{\psi -\psi_{ws}}{\psi_{we}-\psi_{ws}})\right];\;when\;(\psi )\;\;\geq {(\psi }_{ws})$$ (5)

where ${(\psi }_{ws}$) is suction at water saturation point (suction value at which point the soil pores are completely saturated). ${(\theta }_{ws}$) is related to water content at saturation, $\left(\theta_{we}\right)$ is water content at water entry point, while (θ) is calculated water content corresponding to a particular suction (ψ).

### Proposed Equation

Previous studies such as Satyanaga et al. [35] have shown that SWCC of gap-graded soil should not necessarily always be bimodal. The curves, (particularly the wetting) could also be unimodal, especially in disturbed sample, due to pore size re-distribution and the variation of initial water content [45]. The bimodality could also increase when the macropores are dominant, as the percentage of fine content in the sample decreases as well. However, to accurately predict the wetting curve entirely from the drying curve (which is often bimodal), this work assumes a bimodal hysteretic curve.

The derivation of the proposed model relies on the following assumptions.

i. That the shape of the SWCC closely resembles the graph of exponential function, therefore the equation is derived based on equating a normalized water content with the reciprocal of exponential function.
ii. No volume change in the soil sample has been considered.
iii. All the variables of the bimodal drying SWCC from which the bimodal wetting SWCC would be estimated are known.
iv. Hysteresis exists beyond the suction of 1500 kPa, and that the water entry value is less than the residual suction of the same soil.
v. That there is permanent hysteresis, and that the soil would not recover its initial saturated water content upon re-saturation.
vi. That based on thermodynamic theory, the matric suction under fully saturated condition is 0 kPa, and the maximum suction of 10^6^ kPa is achieved at 0% moisture content [46]. As such, the Fredlund and Xing [47] correction factor is adopted.
vii. That the minimum water content of both curves are equal and the same and the two boundary curves merged at higher suction.

These assumptions were made in order to relate the model to different soils conditions as close as possible. And to provide a true impact on model applicability. For instance, while points i, iii, v, vi and vii are relatively valid at all soil types and conditions, points ii and iv varies with the soil type. Volume change isn’t considered in order to make the proposed model as simple as possible and reduce the number of parameters that usually render such models so cumbersome for application. Likewise, in point iv, although many studies assumes hysteresis of SWCC ceases around wilting point (1500 kPa), laboratory tests in this work and several others in the literature such as Kristo and Rahardjo [33] have shown that the hysteresis indeed exist beyond the wilting point and have significant impact on soil-water interaction at high suction.

**Fig 2:**
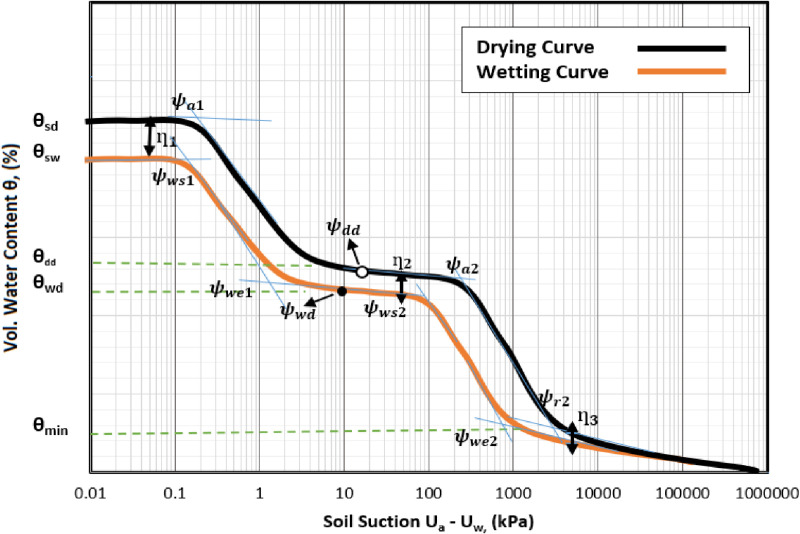
Typical hysteretic bimodal SWCC showing the variables of both curves. where, ${(\psi }_{ws1}$) is suction at water saturation point (when saturation = 100%) for sub-curve 1 (related to wetting curve). ${(\psi }_{ws2}$) is suction at water saturation point (when saturation = 100%) for sub-curve 2 (related to wetting curve). ${(\psi }_{we1}$) is suction at water entry point for sub-curve 1 (related to wetting curve). ${(\psi }_{we2}$) is suction at water entry point for sub-curve 2 (related to wetting curve). ${(\psi }_{wd}$) is suction at wetting delimiting point for the wetting curve. ${(\psi }_{dd}$) is suction at wetting delimiting point for the drying curve. ${(\psi }_{a1}$) is the air entry value of the drying sub-curve 1. ${(\psi }_{a2}$) is the air entry value of the drying sub-curve 2. ${(\psi }_{r2}$) is the residual value of the drying sub-curve 2. ${(\theta }_{ws}$) is related to water content at saturation = 100%.$\left(\theta_{we}\right)$ is water content at water entry point. $\left(\theta_{wd}\right)$ is water content at the delimiting point of the wetting curve. $\left(\theta_{dd}\right)$ is water content at the delimiting point of the drying curve. $\eta_{1,}\;\eta_{2}\;and\;\eta_{3}$ are the hysteresis parameters related to entrapped air in the soil pores.

Overall, the impact of the stated assumptions provided a better estimation capability of the model and conform with the majority of the soil types with varying properties as would be shown in the results section. The assumed bimodal hysteretic SWCC is shown on Fig 2.

Studies such as Durner [48], Rahardjo et al. [49] and Satyanaga et al. [35]; superimposed two unimodal SWCC models to represent a bimodal SWCC. Usually, the first part of the superimposed equations represents the low suction area dominated by the soil’s macropores, while the second part of the equation represents the area of high suction dominated by the soil’s micropores [35,36,50–52].

The significance of a smooth link between two adjacent sub-curves and the continuity of a best-fitting line have been emphasized by Zhao et al. [36]. It gives an accurate position of delimiting point, which indicates the relationship between the dominant pore sizes in soils with dual porosity. Also, experimentally, the delimiting point been an important parameter in the proposed model, it helps in attaining a better performance of the proposed model. In equation (6) and equation (7), values of parameters ***t*** and ***m*** were obtained by reducing the variation between data in the published literature and measured data using the least square method by Zhao et al. [36] who developed a general multimodal SWCC equation. Thirteen number of soils datasets (Table 1) were used to establish the value of the parameters as ***0.3*** and ***5.0*** respectively.

**Table 1:** Summary of bimodal soil data used in the parametric study and calibration process reproduced from Zhao et al. [36].

	Soil type	qs_1_/S_e_	y_a1_ (kPa)	y_a2_ (kPa)	RMSE	R^2^ (%)	Source
**1**	Mature granite residual soil	0.41	1.10	352	0.022	99.28	[53]
**2**	Tropical clayey silt soil	0.41	1.10	36.6	0.007	99.62	[54]
**3**	Granite residual soil	0.32	0.25	50	0.006	99.28	[55]
**4**	Hong Kong saprolite soil	0.33	1.10	13	0.012	98.41	[35]
**5**	Jurong formation, residual soil	0.24	23.9	253	0.003	98.63	[49]
**6**	Compacted silty sand I	0.34	6.00	35	0.011	99.03	[35]
**7**	Pelletized diatomaceous earth	1.00	0.0001	46	0.018	99.43	[39]
**8**	Residual, highly collapsible clay	1.00	5.27	17.53	0.037	98.55	[53]
**9**	Compacted silty sand II	1.00	12.0	30	0.002	99.55	[56]
**10**	Compacted silty sand III	1.00	0.978	31.61	0.002	99.34	[56]
**11**	Colluvial soil	0.50	3.00	630	0.002	96.54	[51]
**12**	Sandstone	0.46	1.20	900	0.001	97.20	[51]
**13**	Compacted silty sand IV	1.00	10.0	45	0.0012	99.72	[56]

Parameter (***t***) is an exponential decay constant, whose value determines the steepness of the curve. Based on the parametric study by Zhao et al. [36], **5.0** is set as a value of parameter **(t)**. In this study, the values were adjusted to range between ***4.5*** and ***5.0*** for greater fitting and better performance of the proposed model. The extreme range of ***4.5–5.0*** represents a model performance ranging from dense tropical clay to residual sand. A value which may provide a mean representation for all soil types and does not temper with overall performance of the model should then be proposed, and therefore, ***4.75*** is adopted.

Therefore, upon incorporating the proposed value of (t), and superimposing the general unimodal equation [Eq. (2)], the equation for bimodal drying SWCC would be;


$$\theta (\psi )=\left\{\theta_{\min}+\left(\theta_{sd}-\theta_{dd}\right)\exp\;[-4.75\;\left(\frac{\psi -\psi_{a1}}{\psi_{dd}-\psi_{a1}}\right)+\;\left(\theta_{dd}{\;-\;\theta }_{\min}\right)\exp\;\left[-4.75\;\left(\frac{\psi -\psi_{a2}}{\psi_{r2}-\psi_{a2}}\right)\right]\;\right\}$$ (6)

And the bimodal wetting SWCC equation would be;



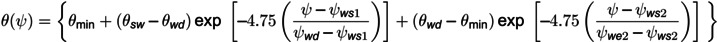

(7)


where:

Subscripts [1] and [2] represent the sub-curves [1] and [2] respectively, relating to the bimodal curve with the macropores and micropores distribution of the dual porosity soil.

$\theta_{sw}$ is the saturated water content of wetting curve at the beginning of sub-curve 1

$\theta_{wd}$ is the water content at the delimiting point between the two sub-curves

$\theta_{\min}$ is water content corresponding to highest laboratory recorded suction

${\;\psi }_{ws1}$ and ${\;\psi }_{ws2}$ are water saturation values related to macropores and micropores of the two sub-curves respectively.

$\psi_{we2}$ is the water entry value of the soil.

$\eta_{1}$, $\eta_{2}\;$ and $\eta_{3}$ in Fig 2 are hysteresis parameters related to the effect of entrapped air in the soil’s pores

To obtain a smooth transition between the two sub-curves of any multimodal SWCC, a wetting delimiting point ($\psi_{wd}$) in Eq. (8) is used, representing a transition point that marks the minimum pore of macropores or the maximum pore of micropores of the soil.


$$\psi_{wd}=\;\psi_{ws1}^{m}*\psi_{ws2}^{1-m}$$ (8)

The (***m***) in Eq. (8) is a parameter of curves delimiting point related to hydration rate at the transition area, where the wetting curve is adjusting to the changes from initial drying condition. Delimiting point is calculated based on the values of wetting saturation points of the two sub-curves. It is related to the position of the two saturation points of the two curves and is controlled by the slope of the sub-curve 1. The proposed model is sensitive to the value of constant (***m***). Increasing the value of (***m***) more than ***0.3*** decreases the value of delimiting point, leading to more steepness of slope of sub-curve 1. Higher value of (**m**) beyond **0.3** pushes the curve to the left and reduces the performance of the model. Whilst, if the value reduces below ***0.3*** the curve would shift forward towards the drying curve and reduce the hysteresis loop. Therefore, the value of ***0.3*** is the best alternative for parameter (***m***) as applied to different soil types.

The variables of the bimodal drying curve needed for the accurate estimation of the wetting bimodal curve are;

i. The first air entry value of sub-curve 1 ($\psi_{a1}$),
ii. The second air entry value of sub-curve 2 ($\psi_{a2}$),
iii. Residual suction of the first sub-curve ($\psi_{r1}$),
iv. Residual suction of the second sub-curve ($\psi_{r2}$),
v. The saturated water content of sub-curve 1 ($\theta_{sd}$),
vi. The water content at the delimiting point of the two sub-curves ($\theta_{dd}$)
vii. Residual moisture content of sub-curve 2 ($\theta_{\min}$)

There are six [6] parameters of bimodal wetting SWCC [Eq. 7] to be estimated from the bimodal drying SWCC [Eq. 6]. These parameters are saturated water content ($\theta_{sw})$; water content at delimiting point of the two sub-curves ($\theta_{wd})$; residual water content of sub-curve 2 ($\theta_{\min})$; water saturation value of sub-curve 1$\;{(\psi }_{ws1})$; suction at the delimiting point of the two sub-curves ${(\psi }_{wd}$); and water entry value of the sub-curve 2 $\left(\psi_{we2}\right)$.

**Fig 3:**
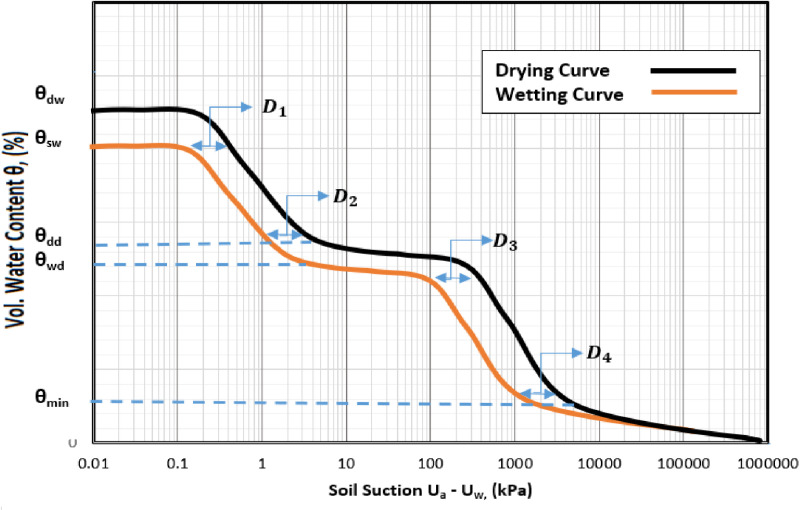
Typical hysteretic bimodal SWCC showing the distance between the curves. D_1_ is the distance between air-entry value of drying sub-curve 1 and water saturation value of wetting sub-curve 1. D_2_ is the distance between residual suction of drying sub-curve 1 and water entry value of sub-curve 1. D_3_ is the distance between air-entry value of drying sub-curve 2 and water saturation value of wetting sub-curve 2. D_4_ is the distance between residual suction of drying sub-curve 2 and water entry value of sub-curve 2. With both curve’s wetting and drying delimiting points (d_dw_ and d_dd_) between D_2_ and D_3_ respectively.

To estimate the bimodal wetting SWCC from the bimodal drying SWCC, a scaling method as utilized by Pham et al, [20] could be used. In this method, the distance between the two curves and their slopes’ ratio is used. The distance between the drying and wetting curves could be adopted as an average of D_1_, D_2_, D_3_ and D_4_ from Fig 3.

The 6 parameters to be used for the estimation could be obtained using equations (9) – (14).


$$Wetting\;saturation\;point\;of\;sub-curve\;1\;(\psi_{ws1})\;\psi_{ws1}=Xa1\;.\;\psi_{a1}$$ (9)

$$Wetting\;saturation\;point\;of\;sub-curve\;2\;\left(\psi_{ws2}\right){\;\psi }_{ws2}=Xa2\;.\;\psi_{a2}$$ (10)

$$Water\;entry\;point\;of\;sub-curve\;2\;\;\left(\psi_{we2}\right)\;\psi_{we2}=Xr2.\;\psi_{r2}$$ (11)

$$Saturated\;water\;content\;of\;the\;wetting\;curve\;\left(\theta_{sw}\right)\;\theta_{sw}=\eta_{1}\theta_{sd}$$ (12)

$$Water\;content\;at\;the\;delimiting\;point\;of\;sub-curve\;\left(\theta_{mid}\right)\;\theta_{wd}=\eta_{2}\theta_{dd}$$ (13)

$$Water\;content\;corresponding\;to\;highest\;laboratory\;recorded\;suction\;\theta_{wmin}=\eta_{3}\theta_{dmin}$$ (14)

To estimate the wetting curve using [Eq. 7] from the drying curve [Eq. 6], the hysteresis parameters (*X*_*a1,*_ *X*_*a2,*_ and *X*_*r2*_) in equation (9)–(14) need to be determined first.

Generally, soil moisture comprises of capillary and adsorbed water. In the area of lower suction, characterized by inter-aggregate pores, capillary forces are lower as compared to the intra-aggregate pores where the forces are high. Studies such as Liu et al. [45] linked the effects of soil pore structure to the hysteresis of SWCC through relating the initial water content and dry density from which the SWCC samples were prepared. The finding revealed that the soil dry density has significant impact on the behavior of the hysteresis loop particularly in the low suction region characterized by bigger soil pores.

Soil density impacts the hysteresis loop due to a number of factors that are influencing soil pore structure. These factors include variation of void ratio and change in pores orientation, different capillary pressure due to different pore sizes and the overall soil ability to retain or expel moisture. Soils with bigger particles are mostly related to higher density and bigger pores. In essence, soils with these higher densities characterized by bigger pores experience low capillary forces, making it easier for moisture to escape into gas phase due to local vapor pressure. The relationship between soil density and change in soil moisture and its hysteresis has been explored in this work.

Six hysteretic SWCC for laboratory engineered soils with varying dry densities were conducted. The difference of saturated volumetric water content of their initial drying curves and the boundary wetting curves due to the influence of entrapped air were studied. An empirical relationship based on the results is used to propose an equation for those entrapped air parameters ($\eta_{1}\;and\;\eta_{2})$ as follows;


$$\left\{\begin{array}{c} \eta_{1}=-3.4943{\left(\rho_{d}\right)}^{3}+15.463{\left(\rho_{d}\right)}^{2}-22.765\left(\rho_{d}\right)+12.144;when\;\rho_{d}\;\geq 1.32\\ else;\eta_{1}=1 \end{array}\right\}$$ (15)

$$\eta_{2}=-4.6898{\left(\rho_{d}\right)}^{3}+21.322{\left(\rho_{d}\right)}^{2}-32.238\left(\rho_{d}\right)+17.163$$ (16)

$$\eta_{3}=1\;(Assumed\;for\;all\;soil\;types\;since\;\theta_{wmin}=\theta_{dmin})$$ (17)

The parameters *X*_*a1,*_ *X*_*a2,*_ and *X*_*r2*_ are equally not variables of the drying curve, therefore must be separately determined before they are used in equation (9) to equation (11). The ratio between slopes of the drying and wetting sub-curves (SR) and the distances between the curves (DC) would be used to determine the parameters X_*a1,*_ *X*_*a2,*_ and *X*_*r2*_ in equation (9)–(11).


$$Slope\;of\;the\;first\;drying\;sub-curve\;(SLd1)=\;\frac{\theta_{sd}-\;\theta_{dd}}{{log\psi }_{r1}-\;\log\psi_{a1}}=\frac{\theta_{sd}-\;\theta_{dd}}{log\;\frac{\psi_{r1}}{\psi_{a}1}}$$ (18)

$$Slope\;of\;the\;second\;drying\;sub-curve\;(SLd2)=\;\frac{\theta_{dd}-\;\theta_{\min}}{{log\psi }_{r2}-\;\log\psi_{a2}}=\;\;=\;\frac{\theta_{dd}-\;\theta_{\min}}{log\;\frac{\psi_{r2}}{\psi_{a2}}}$$ (19)

$$Slope\;of\;the\;first\;wetting\;sub-curve\;(SLw1)=\frac{\theta_{sw}-\;\theta_{wd}}{{log\psi }_{we1}-\;\log\psi_{ws1}}=\frac{\theta_{sw}-\;\theta_{wd}}{log\;\frac{\psi_{we1}}{\psi_{ws}1}}$$ (20)

$$Slope\;of\;the\;second\;wetting\;sub-curve\;(SLw2)=\frac{\theta_{wd}-\;\theta_{\min}}{{log\psi }_{we2}-\;\log\psi_{ws2}}=\frac{\theta_{wd}-\;\theta_{\min}}{log\;\frac{\psi_{we2}}{\psi_{ws2}}}$$ (21)

$$The\;Slope\;Ratio\;(SR1)\;of\;the\;first\;sub-curves\;=\;\frac{{SL}_{d1}}{{SL}_{w1}}\;=\frac{\frac{\theta_{sd}-\;\theta_{dd}}{log\;\frac{\psi_{r1}}{\psi_{a}1}}}{\frac{\theta_{sw}-\;\theta_{wd}}{log\;\frac{\psi_{we1}}{\psi_{ws}1}}}=\;\frac{\theta_{sd}-\;\theta_{dd}}{\theta_{sw}-\;\theta_{wd}}\;\frac{log\;\frac{\psi_{we1}}{\psi_{ws}1}}{log\;\frac{\psi_{r1}}{\psi_{a}1}}$$ (22)

Let 𝛂_i_ = log $\frac{\psi_{ri}}{\psi_{ai}}$; $\theta_{1}$= $\frac{\theta_{sd}-\;\theta_{dd}}{\theta_{sw}-\;\theta_{wd}}\;\;$and substituting into [22]


$$(SR1)=\theta_{1}\left(1\;+\;\frac{\log\;\frac{X_{r1}}{X_{a1}}}{\log\;\frac{\psi_{r1}}{\psi_{a}1}}\right)=\theta_{1}\left(1\;+\;\frac{\log\;\frac{X_{r1}}{X_{a1}}}{\alpha_{1}}\right)$$ (23)

$$\alpha 1\left(\frac{SR1}{\theta_{1}}\;–\;1\right)=\log\;\frac{X_{r1}}{X_{a1}}$$ (24)

$$\frac{X_{r1}}{X_{a1}}=10^{\left[\alpha_{1}\;\left(\frac{SR1}{\theta_{1}}\;–\;1\right)\right]}$$ (25)

In essence, for each sub-curve (i),


$$\frac{X_{ri}}{X_{ai}}=10^{\left[\alpha_{i}\;\left(\frac{{SR}_{i}}{\theta_{i}}\;–\;1\right)\right]}$$ (26)

Therefore, for sub-curves 2,


$$\frac{X_{r2}}{X_{a2}}=10^{\left[\alpha_{2}\;\left(\frac{{SR}_{2}}{\theta_{2}}\;–\;1\right)\right]}$$ (27)

$$\theta_{2}=\frac{\theta_{dd}-\;\theta_{\min}}{\theta_{wd}-\;\theta_{\min}}$$ (28)

The distance between the two boundary curves should be separated into DC_1_ and DC_2_ related to sub-curves 1 and sub-curves 2 based on the two dominant pore-size structures of gap-graded soil.


$$DC1\;=\frac{D1+D2}{2}$$ (29)

$$DC2\;=\;\;\frac{D3+D4}{2}$$ (30)

$$D1\;={log\psi }_{a1}-\;\log\psi_{ws1}=log\;\frac{\psi_{a1}}{\psi_{ws1}}=log\;\frac{1}{X_{a1}}$$ (31)

$$D2\;=\;{log\psi }_{r1}-\;\log\psi_{we1}=log\;\frac{\psi_{r1}}{\psi_{we1}}=log\;\frac{1}{X_{r1}}$$ (32)

$$D3\;={log\psi }_{a2}-\;\log\psi_{ws2}=log\;\frac{\psi_{a2}}{\psi_{ws2}}=log\;\frac{1}{X_{a2}}$$ (33)

$$D4\;={log\psi }_{r2}-\;\log\psi_{we2}=log\;\frac{\psi_{r2}}{\psi_{we2}}=log\;\frac{1}{X_{r2}}$$ (34)

Equation (29) will now become;

$$DC1\;=\;\frac{1}{2}\left(log\;\frac{1}{X_{a1}}+\;log\;\frac{1}{X_{r1}}\right)=\frac{1}{2}\log\left(\;\frac{1}{X_{a1}X_{r1}}\right)$$ (35)

$$DC2\;=\;\frac{1}{2}\left(log\;\frac{1}{X_{a2}}+\;log\;\frac{1}{X_{r2}}\right)=\frac{1}{2}\log\left(\;\frac{1}{X_{a2}X_{r2}}\right)$$ (36)

Eq. (35) and (36) can be re-arranged into;

$$X_{a1}X_{r1}=\;10^{-2DC1}$$ (37)

$$X_{a2}X_{r2}=10^{-2DC2}$$ (38)

Therefore, based on the trend of [37] and [38], general equation for variable (x_i_);


$$X_{ai}X_{ri}=10^{-2DCi}$$ (39)

Putting together [27] and [39]

$$Xa1\;=\;\;10^{\left[-DC1\;–\;\;0.5\alpha \;\left(\frac{SR1}{\theta_{1}}\;–\;1\right)\right]}$$ (40)

$$Xxa2\;=\;10^{\left[-DC2\;–\;\;0.5\alpha \;\left(\frac{SR2}{\theta_{2}}\;–\;1\right)\right]}$$ (41)

$$Xr1\;=\;\;10^{\left[-DC1\;+\;0.5\alpha \;\left(\frac{SR1}{\theta_{1}}\;–\;1\right)\right]}$$ (42)

$$Xr2\;=\;\;10^{\left[-DC2+\;\;0.5\alpha \;\left(\frac{SR2}{\theta_{2}}\;–\;1\right)\right]}$$ (43)

Eq. (40) – (43) have shown that, if distances between the sub-curves and their slopes ratio is known, the variables (*X*_ai_ and X_ri_) could be calculated. Pham et al. [20] provided different values of DC and SR of various soil types as summarized in Table 2.

**Table 2:** Slope ratio and distances between curves for different soil types [20].

Soil type	Slope Ratio (SR)	Distance between Curves (DC)
Sand	2.0	0.20
Sandy Loam	2.5	0.25
Silt Loam and Clay Loam	1.5	0.50
Compacted Silt and Compacted Sand	1.0	0.35

It could be noted that the slope ration (SR) proposed by Pham et al. [20] in Table 2 are generally greater than 1, except for compacted silt and sand. This would lead to positive values of the equations of exponents in X_a_ and X_r_, as against the negative values they should be. Also, the proposed values could not be fully applicable to bimodal soil. To get around this, Satyanaga et al. [57] suggested that SR and *DC* proposed by Pham et al. [20] be taken as a range not a single value, and be applicable to all soil types.

The slope ratio and slope distances proposed by Pham et al. [20] are based on soil types, and these could give a very conservative and wide range of values that may not represent the actual soil’s condition. There should then be values proposed based on actual soil property, which is unique according to soil condition. As such, a new set of values for X_a1,_ X_a2_ and X_r_ applicable for gap-graded soils and bimodal SWCC are therefore suggested in this work in Table 3.

**Table 3:** Suggested values of X_a1,_ X_a2_ and X_r_ according to soil dry density.

Dry Density $\rho_{d}$ (g/cm^3^)	X_a1_		X_a2_		X_r2_	
Value	Range	Absolute	Range	Absolute	Range	Absolute
1.30–1.40	0.59 - 0.48	0.54	0.93 - 1.0	0.97	0.75 - 0.63	0.69
1.41–1.50	0.48 - 0.42	0.45	0.83 - 0.93	0.87	0.63 - 0.53	0.57
1.51–1.60	0.42 - 0.38	0.40	0.72 - 0.83	0.76	0.52 - 0.46	0.49
1.61–1.70	0.37 - 0.30	0.33	0.62 - 0.71	0.67	0.45 - 0.37	0.42
1.71–1.80	0.30 - 0.25	0.28	0.43 - 0.62	0.53	0.37 - 0.29	0.33
1.81–1.90	0.25 - 0.19	0.22	0.42 - 0.35	0.38	0.29 - 0.18	0.25
1.91–2.00	0.19 - 0.11	0.15	0.33 - 0.15	0.21	0.18 - 0.09	0.13

Studies such as Najdi et al. [58] and Liu et al. [45] have shown that soil pore structure is the main attribute that control SWCC hysteresis. The pore structure (size, connectivity and distributions) dictates the amount of moisture the pore could retain, or the suction needed to extract moisture from the pore. Meanwhile, soil density is the fundamental soil property controlling how soil particles are packed together and consequently determine the pore size. Therefore, linking soil density to the overall nature of the hysteresis loop is relevant and not far off. The impact of the soil dry density on the hysteresis parameters (X_a1,_ X_a2_ and X_r_) is equally explored based on soil dry densities, in the same way entrapped air parameters ($\eta_{1}\;and\;\eta_{2})$ were obtained, and corresponding ranges and optimal values are proposed in Table 3.

Alternatively, the following equations can be used for soils with dry density outside of the range of the table.


$$X_{a1}=-0.6296(\rho_{d})+1.3807$$ (44)

$$X_{a2}=\;-0.4566{\left(\rho_{d}\right)}^{2}+0.3389(\rho_{d})+1.3582$$ (45)

$$X_{r2}=-0.862\;(\rho_{d})+1.8425$$ (46)

### Evaluation of the proposed equation

A mathematical model to estimate wetting bimodal SWCC entirely from a drying bimodal SWCC has been proposed. The performance of the model therefore needs to be evaluated, and a statistical method is used. The Root Mean Square Error (**RMSE**) and Coefficient of Determination (**R**^**2**^) are used to evaluate the performance of the proposed equation in comparison to the laboratory measured data of the wetting curves.


$$RMSE\;=\;\sqrt{\left(\frac{\sum_{i=1}^{n}{\left(x_{li}-x_{mi}\right)}^{2}}{N}\right)}$$ (47)

RMSE denotes a sum of the square difference between measured data and an estimated model. A model is perfect if its RMSE is zero. Whilst the other assessment criterion is coefficient of determination (R^2^) shows the proportion of variation of the dependent variable that is predictable from independent variable(s). A proposed model perfection is gauged by how close its R^2^ approaches 1.


$$R^{2}\;=1-\;\left(\frac{\sum_{i=1}^{n}{\left(x_{li}\;–\;x_{mi}\right)}^{2}}{\sum_{i=1}^{n}{\left(x_{mi}\;–\;\frac{1}{N}\sum_{i=1}^{n}\left(x_{mi}\right)\right)}^{2}}\right)$$ (48)

The performance of this proposed model based on RMSE and R^2^ are discussed and shown in the results discussion section.

## Laboratory tests carried out in this study

### Soils Properties

A proposed equation needs to be validated with an experimental dataset to evaluate its performance. Hysteretic SWCC data for different soil types is needed to evaluate the proposed model from this study. Due to high cost and difficulties associated with measurement of SWCC under wetting process there is a limitation of data availability in the literature. A search for SWCC data which are both hysteretic and bimodal provides a scant result. Therefore, additional effort must be made to obtain such datasets that still cover a wide range of soil types to validate the model.

To achieve this, three groups of a hysteretic SWCC dataset were sought and used. First group composed of a complete hysteretic bimodal SWCC from a natural soil obtained from the literature in Najdi et al. [58]. The second is from a hysteretic SWCC dataset of a natural soil obtained from Esil region of Astana, Kazakhstan. To cover a reasonable range of soil properties and obtained enough dataset for the model validation, ten other laboratory-engineered soils were made and used as third group in which results of six are used in proposing an empirical relationship between dry density and hysteresis parameters, and the remaining four are used in the proposed model validation. The engineered soils were obtained by mixing silty sand and inert coarse kaolin at different compositions to obtain additional hysteretic bimodal SWCCs. In total, six soil samples, two from natural soils (NS1 and NS2), and four from laboratory-engineered soils (LS1, LS2, LS3 and LS4), were used to validate the proposed equation. The target is to perform the validation with soil samples having a wide range of properties in order to make the model widely applicable.

Sample for the natural soil (NS1) was prepared using Agropolis clay having low plasticity, obtained from Barcelona, Spain [58]. The clay material properties are shown in Table 4. Its optimum moisture contents and maximum dry densities were obtained, from which the SWCC samples were prepared. The sample for the drying measurement was prepared using 1.69 g/cm^3^, while the sample for the wetting measurement was prepared using 1.50 g/cm^3^. The SWCC tests were conducted using multiple methods of combining the Northumbria High Capacity Tensiometer (N-HCT) and Dewpoint Potentiometer (WP4C).

**Table 4:** Index and other properties of the soils used in validating the proposed equation.

Soil Name	LS1	LS2	LS3	LS4	NS1	NS2
**Soil Type**	**Engineered Soil for laboratory testing used this study**	**Engineered Soil for laboratory testing used this study**	**Engineered Soil for laboratory testing used this study**	**Engineered Soil for laboratory testing used this study**	**Natural Soil for laboratory testing used this study**	**Natural Soil from literature in Najdi et al. [58]**
**USCS**	**CL**	**CL-ML**	**SC**	**SM**	**SM**	**CL**
**Sand (%)**	**10.0**	**40.0**	**60.0**	**90.0**	**85.97**	**48.3**
**Silt (%)**	**85.0**	**57.0**	**38.0**	**9.5**	**7.55**	**42.1**
**Clay (%)**	**5.0**	**3.0**	**2.0**	**0.5**	**6.48**	**9.6**
**OMC (%)**	**20.0**	**12.0**	**10.5**	**9.8**	**13.5**	**16.0**
**MDD (g/cm**^**3**^)	**1.61**	**1.83**	**1.93**	**2.02**	**1.90**	**1.50**
**Liquid Limit (%)**	**42.5**	**27.5**	**23.0**	**17.7**	**39.6**	**29.0**
**Plastic Limit (%)**	**24.5**	**17.2**	**13.4**	**11.7**	**19.3**	**17.0**
**Plasticity Index**	**18.0**	**10.3**	**9.6**	**6.0**	**20.3**	**12.0**
**Specific Gravity**	**2.54**	**2.60**	**2.61**	**2.66**	**2.50**	**2.70**

Soil sample (NS2) is a brownish non-uniform sample obtained along Kabanbay Batyr, in Esil district of Astana, Kazakhstan. The sample was dried at room temperature, pulverized and sieved with No.10 (2 mm) ASTM sieve. A standard proctor compaction test was conducted, and its optimum moisture content and maximum dry density were obtained. 97% dry of optimum was used for SWCC sample preparation. The drying and wetting SWCC tests on the samples were conducted using the combination of High Suction Polymer Sensor (HSPS), and Dewpoint Potentiometer (WP4C).

The soil samples (LS1, LS2, LS3 and LS4) are laboratory-engineered. Sample LS1 composed of 90% kaolin and 10% sand (85% fine content), LS2 composed of 60% kaolin and 40% sand (57% fine content), LS3 composed of 40% kaolin and 60% sand (38% fine content), while LS4 composed of 10% kaolin and 90% sand (10% fine content). The compositions are chosen to obtain a wider range of engineering properties. Engineered soil is chosen for a number of reasons. Satyanaga et al. (2013), Satyanaga and Rahardjo (2019) and Zhao et al. [36] all have indicated that a sand-kaolin mixture, compacted at the dry of optimum would likely produce a bimodal SWCC. The second reason is that the inert course kaolin used in this study is of uniform gradation and does not yield excessive volume change, as such there is high chance of controlling the mix properties. Table 4 shows the overall engineering properties of the soils used in study.

### Index properties tests

All the index properties tests of soil samples in this study are based on procedures described in American Society for Testing and Materials. The soils were classified according to Unified Soil Classification System described by ASTM-D2487-17 [59]. Particle Size Distribution conducted according to ASTM-D6913M-17 [60], the standard proctor compaction tests based on ASTM-D698-12 [61].

### SWCC tests

Both the hysteretic (drying and wetting) SWCC measurement of the soil samples were performed using two separate methods and the results joined together. The first part are conducted using high suction polymer sensor (HSPS) as described by Liu et al. [62] and Liu et al. [45] in which low suctions between (0kPa - 1500kPa) are measured. Whilst the high suction (1500kpa – 300Mpa) were measured using dewpoint potentiometer (WP4C) for all the samples, based on the procedures as described in Leong et al. [63]. Measurements of the low suction were conducted using HSPS because the procedure is faster than compared with the measurement using the conventional methods of measuring low suction such as filter paper, pressure plates and/or Tempe cell. The HSPS used in this study is developed using a synthesized polyacrylamide (PAM), with a varying degree of cross-linking, using ultraviolent polymerization Liu et al. [62]. These specific PAM based tensiometers have better stress relaxation than NaPA (sodium polyacrylate) based. It also has better attribute of maintaining constant pressure for a long period without the need for correction due to pressure decay rate. However, with measuring range of highest accuracy between 10kPa – 1500kPa, PAM-filled tensiometers measuring range is lower than that of NaPA-filled tensiometers [64].

The calibration of the sensor before each round of measurement involves saturating it in de-aired water to enable the polymer to attain the highest swelling and the corresponding highest pressure recorded to be used in calculating the suction at each stage of the on-going test.

**Fig 4:**
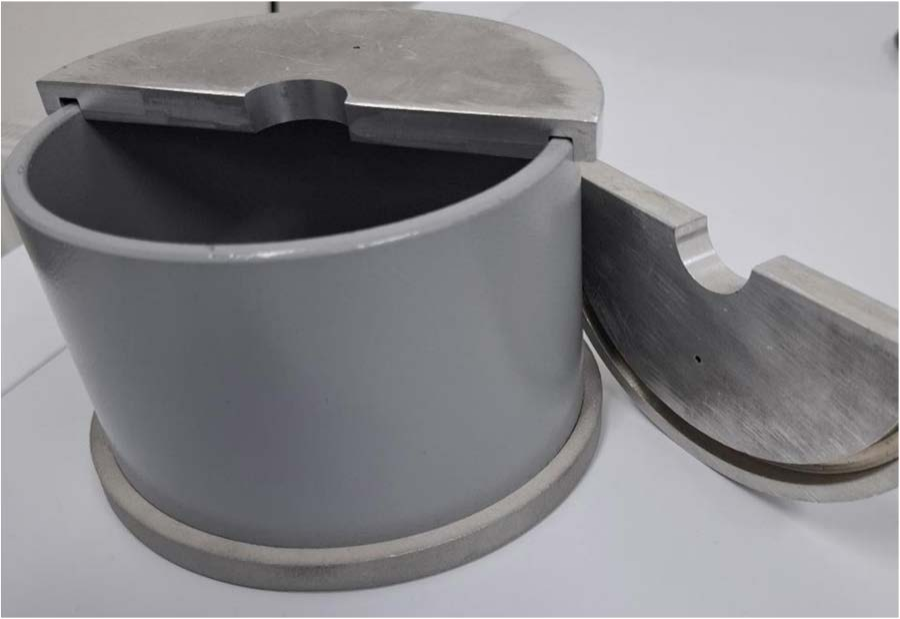
Fabricated Mold for wetting SWCC using HSPS.

Due to a lack of reported procedure to measure wetting SWCC using HSPS in literature, a specialized mold shown in Fig 4 is designed, and fabricated for use in this work is utilized. The cylindrical mold has 120 mm external and 110 mm internal diameters, and with 70 mm height. The cylinder hosting the soil sample is sandwiched between two lids, a bottom lid with several holes to allow for sample saturation from the bottom. And the top lid with central hole to accommodate the sensor, between two other small holes made for sample periodic wetting from top.

### Drying SWCC

The samples for all the compositions of the engineered soils and the natural soil (NS1) were prepared based on the descriptions outlined in ASTM-D6836-16 [65]. The samples are prepared in the mold at 97% dry of optimum, to increase the chance of bimodal curve. The samples are then subjected to saturation by immersing the mold with the prepared sample in a bowl of water. Periodic measurements of weight were carried out and a graph of sample weight against time is continuously plotted. The process is stopped upon full saturation, indicated when either three successive constant weighs or flat horizontal curve is achieved. Saturation of the sensor equally involves immersing the sensor’s bulb completely in water and the pressure build-up recorded until highest pressure is achieved. This indicates that the polymer within the sensor’s polymer chamber has attained its maximum swelling.

**Fig 5:**
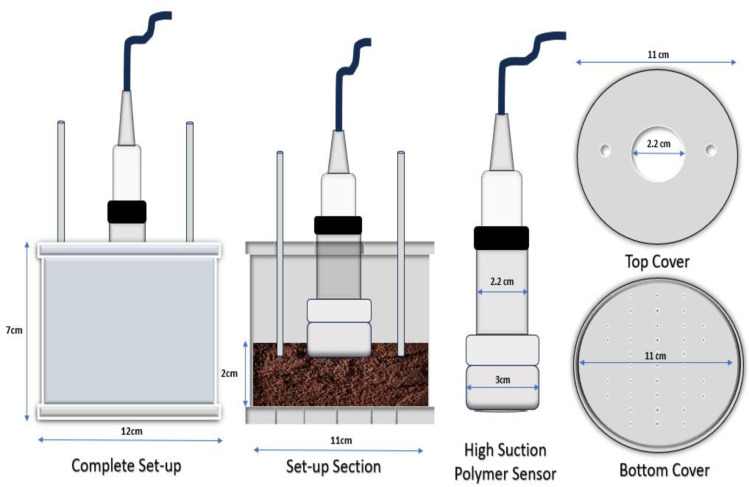
Complete set-up for hysteretic SWCC measurement using high suction polymer sensor (HSPS).

To conduct the dying measurements using HSPS, the sensor is placed onto the sample in the mold with the top lid removed. The sensor bulb buried entirely within the soil sample, with no gap between the sensor and the soil. Formation of such gap may lead to results fluctuation. With the sensor connected to the computer, the whole set-up is placed on to weighing scale having high accuracy to measure moisture loss. The overall initial weight of the set-up is recorded for use in calculating the moisture content at each stage of the test. The testing continues until the maximum suction of around 1500 kPa is recorded. Fig 5 shows a schematic diagram of SWCC testing using HSPS.

**Fig 6:**
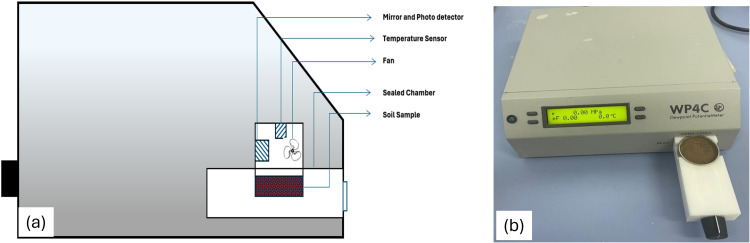
Chilled Mirror Dewpoint Potentiometer (WP4C).

The high suction (1500kPa – around 300MPa) is measures using chilled mirror dewpoint potentiometer (WP4C) shown in Fig 6. The test samples are prepared in a special small container, dried outside of the equipment’s small chamber and cooled. Weights of the samples are recorded both before and after drying for moisture content calculation. The sample is then placed into the equipment’s small chamber for suction measurement using chilled mirror technique. The process has been repeated several times until the suction of around 300MPa is attained.

### Wetting SWCC

The wetting measurement involves covering the mold with the top lid for the entire duration of the test. The top lid has three holes, a central hole of 22 mm diameter, through which the sensor is place and two other smaller holes at the either side of the central hole, through which the sample is periodically wetted. The sample should be completely sealed and no moisture loss due to evaporation allowed. Water is added at intervals, depending on the sample size, while the weight and the change in pressure is recorded.

Moisture content changes within the soil initiate a pressure variation in the pressure chamber and displayed on the connected computer screen for recording. The process is repeated until the full soil saturation is reclaimed. The sample is then dried in an oven for 24 hours. The sample weight is used for back calculating the moisture content at each stage of the test. The moisture contents calculated for both the drying and wetting measurement are merged with the equivalent suction calculated at similar stage and the hysteretic SWCCs are then plotted.

## Results and discussion

### Index properties tests

**Fig 7:**
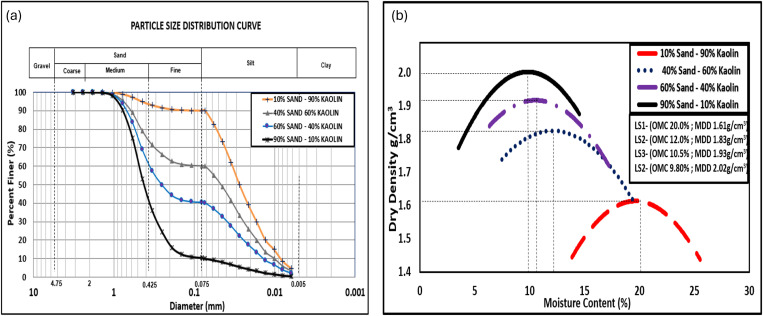
(a) Grain Size Distribution and (b) Standard Proctor Compaction Curves for Engineered Soils.

**Fig 8:**
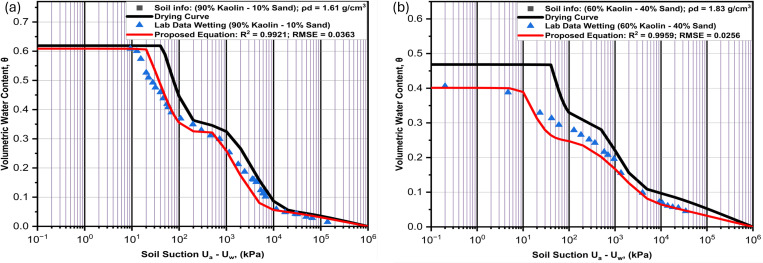
Performance of the proposed model as compared with laboratory results for (a) Soil (LS1) and (b) Soil (LS2).

**Fig 9:**
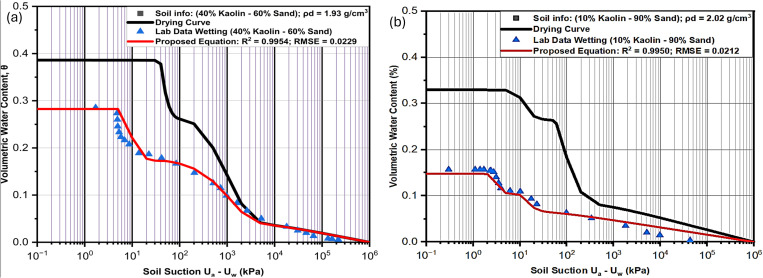
Performance of the proposed model as compared with laboratory results for (a) Soil (LS3) and (b) Soil (LS4).

**Fig 10:**
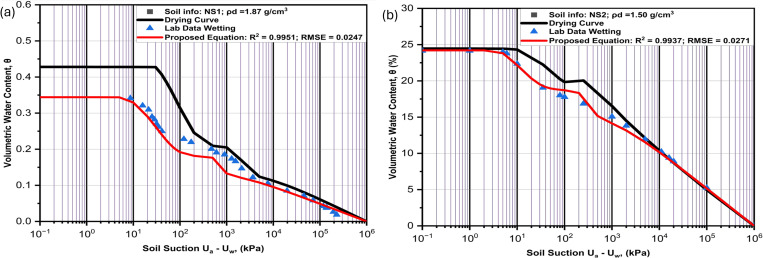
Performance of the proposed model as compared with laboratory results for (a) Soil (NS1) and (b) Soil (NS2).

The index properties tests were conducted on four laboratory engineered samples (LS1, LS2, LS3 and LS4) and one natural soil sample (NS1) from this study. The engineered soil samples were obtained by mixing silty sand and coarse kaolin in different compositions. Soil (LS1) is composed of 90% kaolin and 10% sand. Soil (LS2) is composed of 60% kaolin and 40% sand. Soil (LS3) is composed of 40% kaolin and 60% sand. Soil (LS4) is made up of 10% kaolin and 90% sand. While Soil (NS1) is a natural silty sand obtained from Esil region in Astana, Kazakhstan. The index tests on the samples revealed a wider range of their engineering properties. The particle size distribution tests indicate that all the engineered soils are gap-graded (Fig 7a). Studies by Najdi et al. [58] and Liu et al. [45] shows that initial moisture content and dry densities from which SWCC samples were prepared have direct influence on the bimodality of the SWCC. This is further confirmed in this study, as the choice of 97% dry of optimum moisture content for sample preparation has yielded bimodal SWCCs in all the samples (Fig 8-10). The samples densities and initial moisture contents were chosen to allow covering a wide range of soil types used in model validation, so that the proposed model would be widely applicable. The samples are in the region of 1.61 g/cm^3^–2.02 g/cm^3^ dry densities and initial moisture content of 9.8% − 20.0% (Fig 7).

The results of the engineered soil indicate that an increase in sand percentage in the sample causes an increase in the sample dry density. This is because the sand used in this study has bigger particles, which are heavier (and higher density) than the kaolin used which is mostly silty clay. Due to clay high ability to moisture retention compared to coarser soil [66], the results equally show an increase in moisture content when kaolin contents increased in the four samples. This is also supported by the results of the liquid and plastic limits. Liquid and plastic limits increase with an increase in fine content, due to clay higher specific surface area, the finer material has more moisture retention ability than the coarser sand. Higher fines content also reduces the soil specific gravity, typically because fine particles usually fill the void spaces between the bigger particles, thereby reducing the air pockets and affecting the sample bulk properties which indirectly affect the measure of specific gravity.

### SWCC tests

Several empirical and theoretical studies show that SWCC is the most important element in unsaturated soil analyses, and it is hysteretic in nature. Due to this fundamental significance, its hysteretic feature cannot be ignored any longer in unsaturated soil modeling [67]. Figs 9–10 show the hysteretic SWCC graphs for six soil samples used to evaluate the performance of the proposed equation. Figs 8a, 8b, 9a and 9b are for the four engineered soils having dry densities of 1.61 g/cm^3^, 1.83 g/cm^3^, 1.93 g/cm^3^ and 2.02 g/cm^3^, while Fig 10a and 10b are of natural soils with dry densities of 1.87 g/cm^3^ and 1.50 g/cm^3^ respectively. The laboratory engineered soils were obtained by mixing silty sand and coarse kaolin at varying compositions. It could be noted that the saturated volumetric water content is decreasing as the percentage of fine content is reduced. This is because soils with higher fine content are more liable to retain more moisture in its pores than coarser soils due to clay having a bigger surface area.

It is evident from the curves (Fig 8a,8b, 9a and 9b) that the rate of water retention related to the amount of fine content affects both low suction area and high suction area of the curves. This is indicated by having lower volumetric water content in both sub-curves as the percentage of fine content is reduced. Similarly, the pore moisture is more sensitive to the suction at low suction areas characterized by macropores, showing steeper slope in the first sub-curves (volumetric water content at the beginning of each sub-curve) as compared to more gentler slopes in second sub-curves. This consistence with the Kelvin’s capillary rule indicating that it is much easier for moisture to escape from the soil pores in the area of low suction characterized by bigger pores, compared to a considerable high suction required to extract moisture in the smaller pores. Anandarajah [19] has noted that soils with significant heterogeneity in pore size (macropores surrounded by micropores) exhibit a stronger ink-bottle effect. This phenomenon shows bigger pores (characterized with coarser soil particles) are always emptied first during drying (inkbottle) and is evidently shown in Figs 8a, 8b, 9a and 9b as the air-entry values is higher for soils with more fine content than those with more coarse contents. Also, the water entry values of the wetting curves in all the samples are equally less than the residual suctions of the drying curve, confirming the phenomenon that the micropores are filled first during wetting and last to be emptied during drying.

### Effects of hysteresis

SWCC is hysteretic, researches for long, have linked the phenomenon with several key factors such as entrapped air, ink-bottle effect and routine wetting-drying cycles, and more recently with capillary condensation [68]. Results of all the five samples in this study are hysteretic. Similar to studies such as Satyanaga et al. [35] and Zhao et al. [36], mixture of sand and kaolin yield SWCCs that are bimodal. Likewise in this study, bimodality is identified in all the hysteretic SWCCs. Previous studies (particularly in soil sciences) measure hysteretic SWCC usually up to wilting point (around 1500 kPa). The basic assumption is that plants can no longer extract water beyond 1500 kPa, as such the main area of interest is matric suction below the wilting point. Similar views are held even in the geotechnical field. In this area, it is assumed that air entrapment, which is leading causes of hysteresis, is very limited at this point. Because moisture in bigger pores susceptible to air entrapment would have already been emptied before reaching that suction range. Adsorptive water is equally dominant at high suction and there is limited pore-scale variability to cause ink-bottle effect. These three factors are noted to be responsible for merging of both curves around 1500 kPa. But recent geotechnical studies such as Liu et al. [34] and Kristo et al. [33] indicated that there is indeed a hysteresis loop beyond this point. Capillary condensation, which is not given wide attention compared to the previously stated factors, is the other mechanism causing hysteresis. Capillary condensation influence is majorly around the high suction area. This factor is associated with adsorbed water films on the surfaces of fine-grained particles due to intermolecular forces. It is predominantly controlled by the amount and type of clay mineral content in the soil [69,70]. Capillary condensation is regarded as a responsible factor causing hysteresis beyond the wilting point in this study. This is considering the amount kaolinite clay content in the samples, which according to Israelachvili [71] is responsible for the intricacies related to effects of absorbed water in higher proportion.

In all the engineered soil samples (Fig 8a, 8b, 9a and 9b), the hysteresis loop is wider in the low suction area controlled by macropores. This is related to the effects of entrapped air that filled most of the macropores between the bigger sandy particles during drying. But the envelopes are thinner in the high suction area. The results indicate that soils with higher sand content have difficulty in terms of moisture recovery as compared to samples with higher fine contents. Pharm et al [20] has indicated that the difference of saturated water contents between the boundary drying curve and the boundary wetting curve is in the region of 10%. This can be confirmed by the result of the natural soil (NS1) in Fig 10a. But for laboratory engineered soils, as in (Fig 8a, 8b, 9a and 9b), the difference can extend beyond 10%, depending on the coarse content of the sample. This is because the laboratory engineered soils used in this study do not undergo any wetting and drying cycles like the natural soil, so their drying curve is actually an initial drying curve not boundary drying curve. In general, the hysteresis envelop is wider as the percentage of sand content increases.

With regards to the variables of the curves, the slopes in all the curves are gentler in the samples with higher percentages of the kaolin and steeper in the samples with more sand content. This is in total agreement with several other bimodal SWCC studies. The first and second air entry values of both curves in all the samples are lower in samples with higher sand contents than in samples with more clayey content. The saturated volumetric water contents are equally always higher in samples with more clay content than in those with more sand content. These are all related to more moisture retention ability of clay as compared to sand.

In summary, entrapped air (in the low suction area), capillary condensation (in the high suction area), and ink-bottle effect (across the range of the hysteretic curves) are considered to be the key factors contributing to the hysteresis of samples in this study. Contact angle effect is of negligible effects on hysteresis of soils in this study with the kaolin clay used in the composition having very low angle. As noted by Anandarajah [19], whom considered hysteresis effects on such clayey soils characterized by platelike solids with advancing contact angle between 0–15 would be minimal. And soil samples composed of such clay in favorable proportion may not have cause significant hysteresis to be of noticeable effect. Moreover, Yan et al. [72] noted that the contact angle dynamics is more related to dynamic effects in SWCC rather than hysteretic effects in SWCC. Contact angel dynamics matters with a combination of dynamic effects and hysteresis.

### Model performance

A theoretical model to estimate a bimodal wetting SWCC entirely from a bimodal drying SWCC has been presented and validated in this study. The model relies on parameters (X_a1,_ X_a2,_ and X_r2_) obtained based on the relationship between the distance and the slopes of the hysteretic curves. Parametric study has been conducted to analyses the behavior of these parameters based on change in selected soil properties. The optimal values of these parameters are then proposed based on the dry density from which the SWCC samples are prepared. Six [6] hysteretic bimodal SWCCs were then used for model validation based on the values of the proposed parameters. Table 5] shows the performance of the model in comparison with the laboratory data of the six [6] hysteretic SWCCs used.

**Table 5:** Best fitting parameters for evaluating the performance of the proposed equation.

Soil Name	Soil type	Source	($\rho_{d})$ gcm^-3^	$\psi_{ws1}$ (kPa)		$\psi_{ws2}$ (kPa)		$\psi_{we2}\;(kPa)$		$\theta_{sw}$		$\theta_{wd}$		$\theta_{\min}$		RMSE	R^2^
				Lab^*^	Eqn^*^	Lab^*^	Eqn^*^	Lab^*^	Eqn^*^	Lab^*^	Eqn^*^	Lab^*^	Eqn^*^	Lab^*^	Eqn^*^		
**LS1**	**CL**	Laboratory Engineered Soil used in this study	**1.61**	**16.8**	**19.71**	**529**	**508.11**	**11460**	**9333.0**	**0.609**	**0.609**	**0.329**	**0.325**	**0.065**	**0.065**	**0.0363**	**99.21**
**LS 2**	**CL-ML**	Laboratory Engineered Soil used in this study	**1.83**	**8.5**	**9.14**	**155**	**151.50**	**9160**	**9973.3**	**0.406**	**0.401**	**0.279**	**0.260**	**0.120**	**0.120**	**0.0256**	**99.59**
**LS 3**	**SC**	Laboratory Engineered Soil used in this study	**1.93**	**4.8**	**6.58**	**41.59**	**48.32**	**5230**	**5616.4**	**0.286**	**0.283**	**0.178**	**0.173**	**0.045**	**0.045**	**0.0229**	**99.54**
**LS 4**	**SM**	Laboratory Engineered Soil used in this study	**2.02**	**2.4**	**2.48**	**10.11**	**10.53**	**22.9**	**39.97**	**0.148**	**0.148**	**0.104**	**0.104**	**0.089**	**0.069**	**0.0212**	**99.50**
**NS 1**	**SM**	Laboratory Engineered Soil used in this study	**1.87**	**8.64**	**6.89**	**605.14**	**625**	**1550.54**	**1416.8**	**0.341**	**0.344**	**0.191**	**0.185**	**0.140**	**0.140**	**0.0247**	**99.51**
**NS 2**	**CL**	Natural Soil from literature in Najdi et al. [58]	**1.50**	**5.0**	**4.1**	**280**	**195**	**885.0**	**853.5**	**24.15**	**24.21**	**19.05**	**19.01**	**8.87**	**15.87**	**0.0271**	**99.37**

* Lab = Value from the laboratory data

* Eqn = Value from the proposed model

The model performed very well as compared to the results of laboratory measured data for all the six soil samples. Linking the hysteresis parameters (X_a1,_ X_a2_ and X_r2_) with the dry densities gives a reasonable estimated wetting curve in all the samples. For soil LS1, whose dry density is 1.61g/cm^3^, the proposed model performed well, with R^2^ and RMSE of 99.21 and 0.0363 respectively. In comparing the values of the wetting curve variables, the water saturation value from the laboratory measured data and the estimated value are very close, at 16.8 kPa and 19.71 kPa respectively. Showing a good performance of the model. The trend is quite similar in all the variables of the wetting curve, indicating a significant performance of the proposed model. The only outlier to the trend is the value of ${(\theta }_{\min})$ from the natural soil NS2, where a value 7% moisture content difference is identified between the laboratory measured data and the proposed value. This could be related to the variation of the dry density from which samples were prepared. Because while the drying curve sample was prepared using the dry density of 1.67g/cm^3^, the wetting curve was prepared with 1.50g/cm^3^. In general, the hysteresis of SWCC graphs (Fig 8–10) and the variables comparison (Table 5) show a very good performance of the proposed model for all the soil samples used in the evaluation.

It is worth mentioning that although the model performed well based on the values of the proposed parameters according to influence of dry density to the shape of SWCC. Intricacies surrounding hysteresis of bimodal SWCC require further studies. For example, effects of moisture content and pore size distribution could be explored more rigorously. The model calibration could also be open to improvement particularly with regards to its transition part where the curve is adjusting to the change from initial condition.

**Fig 11:**
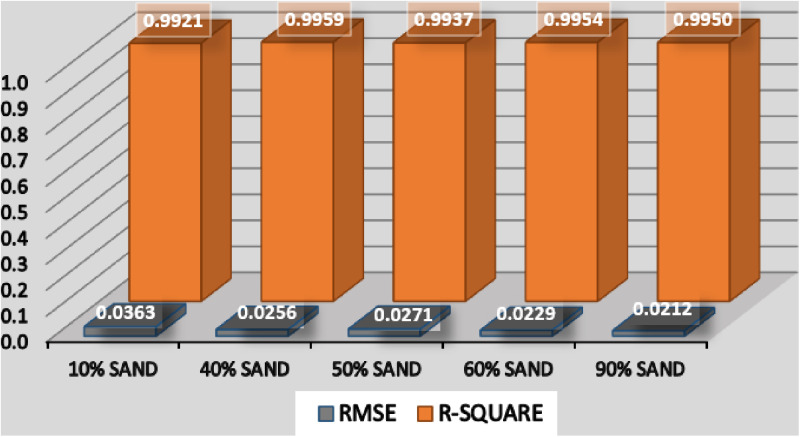
Error analysis based on percentage of sand in a sample.

Based on the values of root mean square error (RMSE) and the coefficient of determination (R^2^), an error analysis for the model is conducted based on percentages of sands in different samples. The error analysis in Fig 11 has shown that irrespective of percentage of coarse or fine content in a given sample, the model performs very well, with all the R^2^ very close to 1 and RMSE close to 0. This has (once again) proved the model’s wide applicability to diverse soil types.

## Conclusions

Soils with two dominant pore size (micropores and macropores) that are characterized by bimodal SWCC are encountered in many natural formations across the globe, and their use in man-made earth structures is common. The majority of failures in earth structures occur during soil wetting, as such accurate analyses of soil mechanical behavior during wetting process is very important. Despite the relevance of wetting SWCC in modelling soil wetting process, researchers often ignore the wetting curve due to the cost, difficulties and longer duration associated with its measurement and use the drying curve. This may lead to inaccurate analyses and designs. An estimation procedure for the wetting curve with the required accuracy and precision to solve those challenges associated with laboratory measurement of the wetting SWCC and help in conducting accurate analyses and designs is lacking in literature. This work then presents a model using scaling approach to estimate bimodal SWCC under wetting process within a short period and with required accuracy. The model development is based on exponential function utilizing the similarity between SWCC and the graphs of exponential function. The model having all of its parameters with physical meaning is validated using laboratory results of hysteretic SWCCs, conducted using the combination of high suction polymer sensor (HSPS) and dewpoint potentiometer (WP4C).

1. SWCC for six soil types (two natural soil and four laboratory engineered soil) with wide range of key properties were used in the model validation. All the SWCCs are hysteretic and bimodal, with the low suction measured with HSPS and the high suction using WP4C.
2. The bimodal curves under wetting process measured in the laboratory were compared with the estimated curves using the proposed model. The results show a significant performance of the proposed model, with the SWCC variables from laboratory measured data and the estimated from the model having very close and similar values.
3. The overall model performance has been evaluated through statistical methods using the coefficient of determination (R^2^) and Root Mean Square Error (RMSE). The evaluation also indicates a good performance of the model, with an average RMSE of 0.0263 and an average R^2^ of 0.9945 for the six soil samples used in the validation.

The study uses the scaling approach for modelling hysteresis of bimodal SWCC. The approach relies solely on the positions of SWCC variables to model the hysteresis of the SWCC. The method entails assumptions that may imposed constraints to the generic attributes of SWCC hysteresis, that the implications of key mechanisms contributing to the general hysteresis of SWCC are not fully taken into account. To get around this limitation, this works relate the scaling parameters (X_a1,_ X_a2_ and X_r2_) with a soil dry density, which is physical attribute that controls the pore size distribution and bimodality of the SWCC, hence the hysteresis of bimodal SWCC. This has given the model more physical meaning than relying solely on the relative positions of the curves from each other in terms of distances between them and their slopes ratios. The study provides a faster approach to obtaining accurate bimodal wetting SWCC entirely from the drying SWCC. It provides the most sought solutions to the challenges associated with the laboratory measurement. This would help with conducting relevant and accurate numerical analyses and designs. The work would also improve modelling hydro-mechanical properties of soil under wetting process significantly and be utilized in practical geotechnical applications.

Moreover, as the data for hysteretic bimodal SWCC (especially the bimodal wetting curve) is very scarce in the literature, data of laboratory test in this work would further enrich the literature for further research use.

Although the model performed very well based on the values of the proposed parameters according to the influence of dry density on the shape of SWCC, intricacies surrounding hysteresis of bimodal SWCC require further studies. For example, effects of moisture content and pore size distribution could be explored more rigorously. The model calibration could also be open to improvement particularly with regard to its transition part. A further potential refinement is suggested to include an additional parameter that could address the occurrence of an outlier in the transition zone of the curve.

Moreover, additional studies related to the effect of volume change could be looked into and incorporated in the model. This meanwhile should not be too extreme, to avoid rendering the model to be too cumbersome and difficult for practical applications.

## Notations

**Table:** 

D _ 1 _ distance between air-entry value of drying sub-curve 1 and water saturation value of wetting sub-curve 1
**D **_**2 **_ distance between residual suction of drying sub-curve 1 and water entry value of sub-curve 1
**D **_**3 **_ distance between air-entry value of drying sub-curve 2 and water saturation value of wetting sub-curve 2
**D **_**4 **_ distance between residual suction of drying sub-curve 2 and water entry value of sub-curve 2.
**d **_**dw **_delimiting point of the wetting sub-curves
**d **_**dd **_delimiting point of the drying sub-curves
**DC **_**i **_distance between sub-curves (i)
**m **parameter of curves delimiting point related to hydration rate at the transition area
**N **number of observable variable
**t **scaling parameter related to curve slope
$\eta_{i,}$ hysteresis parameters related to entrapped air in the soil pores
$x_{li}$ actual value of the dependent variable for observation
$x_{mi}$ predicted value from the regression model for observation
***X ***_***ai, ***_ ***X ***_***r2 ***_ hysteresis parameters related to soil density
SL _di _slope of the drying sub-curve (i)
SL _wi _slope of the wetting sub-curve (i)
**SR**_**i**_slope ratio between sub-curves (i)
𝛂_i_ ratio between residual suction and air entry value
$\rho_{d}$ soil dry density
θ calculated water content corresponding to a particular suction ( ψ )
$\theta_{dd}$ water content at the delimiting point of the drying curve
$\theta_{wd}$ water content at the delimiting point of the wetting curve
$\theta_{sw}$ related to water content at saturation = 100%
$\theta_{we}$ water content at water entry point
$\theta_{wmin}\;$ water content corresponding to highest laboratory recorded suction for wetting curve
$\theta_{dmin}\;$ water content corresponding to highest laboratory recorded suction for drying curve
$\psi_{a1}$ air entry value of the drying sub-curve 1
$\psi_{a2}$ air entry value of the drying sub-curve 2
$\psi_{dd}$ suction at drying delimiting point for the drying curve
$\psi_{r2}$ the residual value of the drying sub-curve 2
$\psi_{wd}$ suction at wetting delimiting point for the wetting curve
$\psi_{we1}$ suction at water entry point for sub-curve 1 (related to wetting curve)
$\psi_{we2}$ suction at water entry point for sub-curve 2 (related to wetting curve)
$\psi_{ws1}$ suction at water saturation point (when saturation = 100%) for sub-curve 1 (related to wetting curve)
$\psi_{ws2}$ suction at water saturation point (when saturation = 100%) for sub-curve 2 (related to wetting curve)
